# Assessment of Climate Change Impacts on Chilling and Forcing for the Main Fresh Fruit Regions in Portugal

**DOI:** 10.3389/fpls.2021.689121

**Published:** 2021-06-23

**Authors:** Helder Fraga, João A. Santos

**Affiliations:** ^1^Centre for the Research and Technology of Agro-Environmental and Biological Sciences, Universidade de Trás-os-Montes e Alto Douro, Vila Real, Portugal; ^2^Institute for Innovation, Capacity Building and Sustainability of Agri-food Production, Vila Real, Portugal

**Keywords:** chilling requirements, heat forcing, Portugal, climate change, apple, pear, plum, orange

## Abstract

Air temperature plays a major role in the growth cycle of fruit trees. Chilling and forcing are two of the main mechanisms that drive temperate fruit development, namely dormancy and active plant development. Given the strong sensitivity of these crops to air temperature and the foreseeable warming under future climates, it becomes imperative to analyze climate change impacts for fruit trees. The fruit sector in Portugal has risen significantly over the last decades, gaining increasing importance both internally and through exports. The present research assesses the impacts of climate change on the chilling and forcing for economically relevant fruit trees in Portugal, namely apples, oranges, pears, and plums. To assess temperate fruit chilling and forcing conditions, the chilling portions (CP) and growing degree-hours (GDH) were computed over Portugal, for the recent-past (1989–2005) and future (2021–2080) periods, following two anthropogenic radiative forcing scenarios (RCP4.5 and RCP8.5). Future climate data were obtained from four regional-global climate model pairs to account for model uncertainties. Bias-correction methodologies were also applied. A spatial analysis over the main regions with PDO “Protected Denomination of Origin” or PDI “Protected Geographical Indication” of origin of each fruit tree was performed. Future projections show a clear decrease in chilling for all regions and fruit types in Portugal. Nonetheless, given the current chilling values in Portugal and the relative importance of chilling accumulation for each fruit type, these changes are more significant for certain varieties of apples than for other types of fruit. Regarding forcing, the future projections highlight an increase in its values throughout the different fruit tree regions in Portugal, which should lead to earlier phenological timings. These changes may bring limitations to some of the most important Portuguese temperate fruit regions. The planning of suitable adaptation measures against these threats is critical to control the risk of exposure to climate change, thus warranting the future sustainability of the Portuguese fruit sector, which is currently of foremost relevance to the national food security and economy.

## Introduction

Temperature is considered the most important atmospheric factor for crop phenological and physiological development. Therefore, climate change is considered a major threat, as it is expected to alter the canopy microclimatic conditions of crops. As an example, future warming should lead to increasing growing season temperatures and decreasing wintertime chilling (Atkinson et al., 2013; Lee and Sumner, 2016), which may significantly affect the currently adopted plant species. This is particularly important taking into account that some crops are the basis for the socio-economic development and livelihood of some regions. Specifically, for the Mediterranean region, which currently hosts some of the most important fruit orchards in the world, a changing climate may represent an important menace to economically-relevant crops (IPCC, 2014; Fraga et al., 2020b). The Mediterranean regions are considered a climate change hotspot, i.e., “a region for which potential climate change impacts on the environment or different activity sectors can be particularly pronounced” (Giorgi, 2006). Hence, it becomes clear that fruit production in the Mediterranean may be particularly vulnerable to climate change (Baldocchi and Wong, 2008; Luedeling et al., 2011), namely in southern Europe (Atkinson et al., 2013).

As a result of a careful long-term plant selection by growers, each type or variety of fruit tree tends to be grown in the most suitable areas within a given region, resulting in a stable site-crop relationship, with the most adequate pedoclimatic conditions to maximize yield and quality parameters (San-Miguel-Ayanz et al., 2016). This relationship may be threatened under climate change scenarios. Depending on the regional strength of the climate change signal, suitability for each fruit/variety may show contrasting developments under future climates. Given that air temperature is a fundamental atmospheric factor influencing fresh fruit growth and development rates, different fruits/varieties have different thermal thresholds for adequate physiological and phenological development, including fruit ripeness (Schwartz and Hanes, 2010; Ikinci et al., 2014). There are usually two main thermal factors influencing fruit development in temperate climatic regions, i.e., chilling and forcing (Ruiz et al., 2007; Benmoussa et al., 2017; Santos et al., 2018). While the onset of vegetative development generally occurs following a chilling period, actual growth and development are driven by forcing (Osborne et al., 2000).

Temperate fruit species commonly require cool enough winters to fulfill their chilling requirements and allow normal stable development and harvests (Legave et al., 2010; Campoy et al., 2011; Darbyshire et al., 2011). In particular, for apple (Severino et al., 2011; Cardoso et al., 2015), pear (Kretzschmar et al., 2011; Hussain et al., 2015), plum (de Carvalho et al., 2010; Ruiz et al., 2018), a period of relatively low temperatures is needed for regular budding (chill accumulation). For the case of citrus, such as oranges, these plants are usually grown in warmer climates, and exposure to cold temperatures (<5°C) may result in chilling injuries (Yuen et al., 1995; Santos et al., 2019). When spring arrives, forcing becomes a much stronger driver of tree phenology than chilling (Luedeling et al., 2013), and a period of warm temperatures (heat accumulation) is needed for adequate blooming and fruit ripening (Arroyo et al., 2013; Drepper et al., 2020; Rodriguez et al., 2020; Cho et al., 2021).

Given the high sensitivity of temperate fruit trees to thermal conditions, it becomes clear that the ongoing global warming may have significant impacts on fruit quality and productivity (Campoy et al., 2011; Luedeling et al., 2011; Benmoussa et al., 2017, 2020; Buerkert et al., 2020; Fernandez et al., 2020). Hence, assessing present and future chill and heat accumulations is essential for identifying the most suitable fruit tree species for a given site, for maintaining economically viable fruit orchards, or for ensuring that detrimental impacts of climate change can be effectively lessened (Gao et al., 2012). Various studies have combined chill and heat indices for studying thermal requirements of different fruit trees or for developing models of phenology (Maulión et al., 2014; Fraga et al., 2015; Guo et al., 2015b; Santos et al., 2017). Nonetheless, a comprehensive analysis of the main fresh fruit orchards in Portugal, and their relationship with chilling and forcing is still incipient.

**FIGURE 1:**
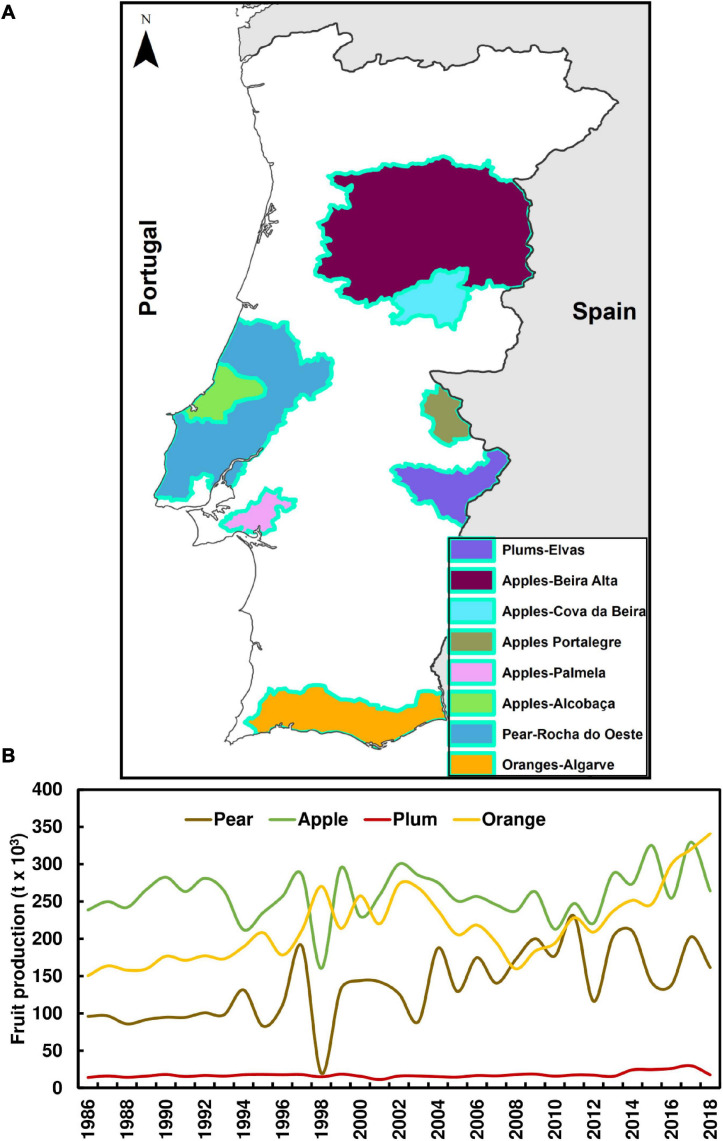
**(A)** Main fresh fruit Denomination of Origin regions over mainland Portugal, including apples, plums oranges, and pears. **(B)** Chronogram of the main fresh fruit produced in Portugal from 1986 to 2018.

In Portugal, fresh fruit crops are particularly important in the socio-economic context of the country, which is known for its high-quality fruit products. Some of the most important regions are illustrated in Figure 1A. Portugal currently has the fourth fruit largest fruit-producing area and is the sixth largest producer in Europe (Eurostat, 2021). This sector is based on an intensive human labor force, which is crucial for the income of several regions in the country. Amongst the most productive fruit species grown in Portugal are oranges, apples and pears. These fruits account for 39, 30, and 19% of the total fruit production in Portugal, respectively (Figure 1B; INE, 2021). The Portuguese climatic characteristics, generally characterized by temperate Mediterranean-type climates (Costa et al., 2016), with moderate temperatures throughout the year and relatively low risk of severe weather (e.g., hailfall, windstorms), are favorable for the development of temperate fresh fruits, such as apples, peaches, pears, amongst others. Over the Portuguese territory, there are several fruit-producing regions, many of which are regulated by law, such as PDO (Protected Denomination of Origin) or PGI (Protected Geographical Indication) regions. According to the EU (2021), “*The differences between PDO and PGI are linked primarily to how much of the product’s raw materials must come from the area or how much of the production process has to take place within the specific region*.” These geographical distinctions identify high-quality products originating from a specific region, with particular environmental conditions, including climate, soils, besides other natural and human factors. In Portugal, there are several PDO or PGI regions linked to the fresh fruit sector.

Bearing in mind the key role played by the fruit sector on the national economy and the threats emerging from climate change, it is important to assess the potential impacts that future climate may have on the fresh fruit sector. By providing insight on future trends over the bioclimatic conditions, these outcomes may support decision-making and stakeholders from the Portuguese fruit sector, as timely planning of suitable adaptation measures helps mitigating future losses and promotes the long-term sustainability of this sector.

The present study aims at analyzing the impacts of climate change on chilling and forcing conditions of fruit orchards in Portugal. As such, the objectives of the present study are five-fold: (1) to compute recent-past thermal conditions over Portugal, using the Safe Winter Chill index, based on chilling portions (CP), and the Safe Heat Forcing index, based on the growing degree hours; (2) to link these thermal conditions to the regions where the main fresh fruit crops are located; (3) to compute future changes of these thermal conditions, using an ensemble of bias-corrected high-resolution climate model simulations; (4) to analyze the interannual variability embedded in future climates, as well as uncertainty linked to climate model simulations; (5) to compare different fresh fruit regions and crops under climate change; and, lastly, (6) to discuss potential adaptation measures.

## Materials and Methods

### Fresh Fruit Regions

In the current study, two types of fresh fruit regions are considered: PDO and PGI. These two types of denomination belong to the European Union (EU) Geographical Indications System, which protects the names of products that originate from specific regions. For the sake of succinctness, these two types of regions are henceforth denominated as fresh fruit regions. Currently, eight fresh fruit regions are considered across mainland Portugal, and consequently eight PDO or PGI regions/products (Table 1). These include: Plums from Elvas PDO; Apples from Alcobaça PGI; Apples—Beira Alta PGI; Apples from Cova da Beira PGI; Apples from Portalegre PGI; Apples—from Palmela PDO; Pears—Rocha do Oeste PDO; Oranges—Algarve PGI. Although many other PDO and PGI regions exist within the country, they are out of the scope of the present study. In order to analyze the spatial distribution of fruit orchards within each region, the COS 2010 (“Carta do Uso do Solo”) was used, which is a land use and land cover (LULC) dataset over mainland Portugal (Source: Direção-Geral do Território, http://www.dgterritorio.pt/). The area for fruits is linked to the different fresh fruit regions (either PDO or PGI) and the subsequent analysis is only performed in areas containing fresh fruit trees based on the COS 2010 LULC classes.

**TABLE 1:** Fresh fruit regions and main fruit varieties grown in Portugal according to the DGADR (Direção-Geral de Agricultura e Desenvolvimento Rural, https://tradicional.dgadr.gov.pt/).

**Region**	**Main variety**
Plums—Elvas PDO	*Rainha Cláudia*
Apples—Alcobaça PGI	*Casa Nova, Golden Delicious, Red Delicious, Gala, Fuji, Granny Smith, Jonagold, Reineta, Pink*
Apples—Beira Alta PGI	*Golden, Gala, Red Delicious, Starking, Jonagold, Granny Smith, Jonared, Reineta*
Apples—Cova da Beira PGI	*Golden Delicious, Red Delicious, Jersey Mac, Bravo de Esmolfe*
Apples—Portalegre Maça PGI	*Bravo*
Apples—Riscadinha de Palmela PDO	*Riscadinha*
Pear—Rocha do Oeste PDO	*Rocha*
Orange—Algarve PGI	*Newhall, Valencia Late*

### Chill and Heat Accumulations

Fresh fruit trees (e.g., apple, pear, and plums) have pre-defined thermal ranges for heat and chill requirements. A specified sum of low temperatures is needed for regular budding, while a period of warm temperatures is needed for adequate flowering and fruit maturation, though these pre-requisites are also largely variety-dependent (De Melo-Abreu et al., 2004; Maulión et al., 2014; Santos et al., 2017). To quantify the chill and heat requirements of fresh fruit trees, several modeling approaches have been developed (Benmoussa et al., 2017). In the present study, to assess fruit chilling and forcing, the CP Dynamic model (Fishman et al., 1987) and the Growing Degree Hours (GDH) model (Anderson et al., 1986; Gu, 2016) were computed. Although other widely used models, such as Chilling Hours or growing degree days (e.g., Matzneller et al., 2014; Morais and Carbonieri, 2015; Spinoni et al., 2015), can be used for this purpose, they are usually outperformed by CP and GDH (Luedeling and Brown, 2011).

Both the CP and the GDH take advantage of an hourly assessment of temperatures. The CP dynamical model is based on an hourly temperature curve, ranging from –16 to 24°C, with an optimum around 4°C, from October to February. Furthermore, the CP also takes into account the counteracting effect of higher temperatures on earlier chilling, which is particularly important in warm climates, such as in Portugal (Ruiz et al., 2007; Luedeling et al., 2009). For GDH, in turn, the hourly thermal accumulation occurs from February to October, with a base temperature of 4°C and critical temperature of 36°C, with an optimum at 25°C (Anderson et al., 1986; Guo et al., 2015a). For calculating both GDH and CP, the R^®^ package “chillR” version 0.70.2 was used (Luedeling, 2013). The time periods for thermal accumulation (CP: October to February; GDH: February to October) were hereby selected to take into account possible future changes in plant development timings (advances/delays) (Guo et al., 2015a).

### Climatic Data

For the recent-past (1989–2005, henceforth baseline period), daily maximum and minimum (2 m) air temperatures were retrieved from the observation-based E-OBS gridded dataset, version 22.0e (Cornes et al., 2018). Data were retrieved on a 0.1° regular grid (∼10 km resolution). Regarding future projections (2021–2080, henceforth future period), simulations generated by four global-regional climate model pairs (GCM-RCM, Supplementary Table 1), under two future scenarios (RCP4.5 and RCP8.5), were retrieved from the EURO-CORDEX dataset (Jacob et al., 2014), and defined on a 0.125° regular grid (∼12.5 km resolution). Aiming to homogenize the baseline and future periods in terms of their spatial resolution, the baseline period data were bi-linearly interpolated to the coarser EURO-CORDEX grid. This allows the computation of both CP and GDH on the same grid and spatial resolution (12.5 km).

Upon this preliminary assessment, the simulated GCM-RCM daily maximum and minimum temperatures were bias-corrected, using E-OBS as a baseline and applying the “Empirical Quantile Mapping” methodology (Cofiño et al., 2017). Lastly, both CP and GDH are then computed for the future period using the bias-corrected data as input in ‘chillR’ (Luedeling et al., 2013). GDH and CP were computed for each GCM-RCM separately and multi-model ensembles were subsequently computed for the future period. For climate change assessments, three sub-periods were considered and compared to the baseline period (1989–2005), i.e., short-term (2021–2040), medium-term (2061–2080), and long-term (2061–2080). To analyze the agreement among the four global-regional climate model pairs, the future outputs were assessed regarding the model ensemble as well as the model standard deviation. Linear trends over the eight fresh fruit regions for the CP and GDH for the future period were also assessed.

Additionally, for baseline and future subperiods, two other metrics were computed: the Safe Winter Chill, SWC (Luedeling et al., 2011)—the minimum amount of CP found in 90% of all years (the 10th percentile); and the Safe Heat Forcing (SHF)—the minimum amount of GDH found in 90% of all years (the 10th percentile). These two metrics are commonly more useful for growers than average CP and GDH (Luedeling et al., 2011), as they provide the risk of not having enough chilling or forcing during an orchard’s lifetime. Henceforth, for the sake of succinctness, the GDH and SHF values are shown in ×10^3^.

The computation of the CP and GDH was performed over the entire Portuguese mainland, using the gridded datasets described above, and subsequent regional assessment was then performed by extracting data for each region. To compute the CP and GDH values over a large spatial extension, such as mainland Portugal, an additional MATLAB^®^ script was developed to automatize this process. The source code can be found in Supplementary Material.

## Results

### Historical Safe Winter Chill and Safe Heat Forcing

**FIGURE 2:**
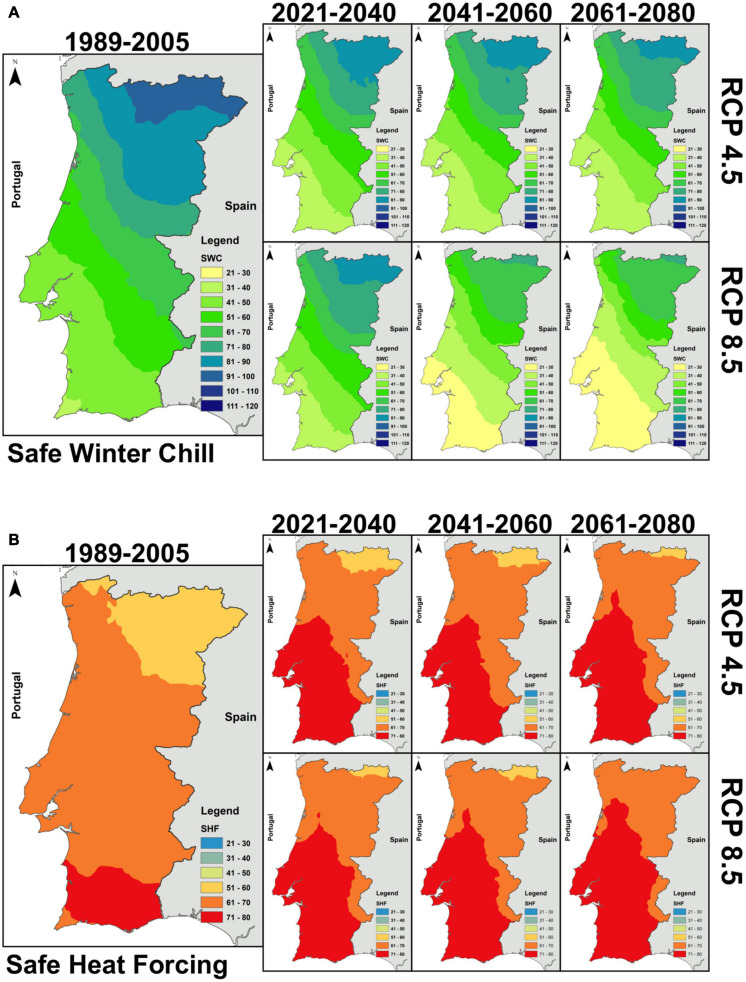
**(A)** Safe Winter Chill index over mainland Portugal for the baseline period (1989–2005) and for three future periods (2021–2040, 2041–2060, and 2061–2080) and two future scenarios (RCP4.5 and RCP8.5). **(B)** Same as panel **(A)** but for the Safe Heat Forcing index, values are shown in ×10^3^.

The spatial patterns of the SWC and SHF for the baseline are shown in Figure 2. In both indices, their patterns show a strong latitudinal gradient, with the warmer areas in the south and the cooler areas in the north. The SWC values throughout the country range from 30 to 40 CP, in the southwestern, to 110, in the northeastern parts of the country (Figure 2A). In effect, values below 40 CP are relatively absent throughout the country, only present in a small area in the south. SHF is commonly greater than 50 GDH (note that GDH values are in ×10^3^) in the inner-most north part of the country, increasing up to 80 GDH in the southern areas (Figure 2B). Values above 80 GDH are currently limited to southern Portugal.

**FIGURE 3:**
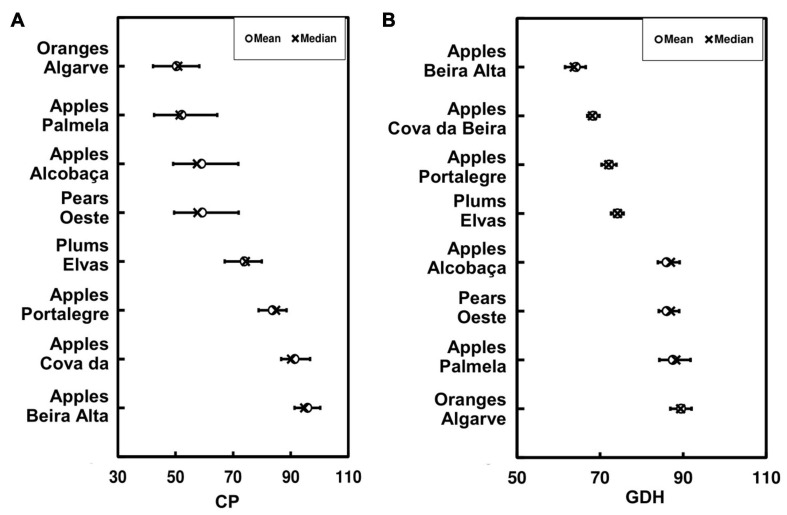
Boxplots representing the chilling portions (CP) **(A)** and growing degree hours (GDH) **(B)** over the selected eight fresh fruit regions. The regional mean (circle) and median (cross) are depicted, as well as the minima and maxima (outer dashes). The regions are ranked from the lowest to the highest mean values of SWC and SHF, respectively. GDH and SHF values are shown in ×10^3^.

A regional assessment of the values of the CP and GDH was also performed over the selected fresh fruit regions (Figure 3). Regarding CP (Figure 3A), the regions that currently have lower CP values are devoted to orange production in “Algarve” (40–60 CP). This was anticipated, as it is known that orange development is not limited by chilling requirements. Surprisingly, apples from “Palmela” also show low CP values (40–65 CP), very similar to those of “Algarve.” These low CP values may prove useful under future warmer climates. Subsequently, apples from “Alcobaça” and pears from “Oeste” show CP values ranging from 50 to 70 CP. The fresh fruit regions with the highest CP values are apple producers in “Cova da Beira” and “Beira Alta,” with values of 85–95 CP. Regarding GDH (Figure 3B), opposite gradients are found, meaning that the regions with the lowest CP have the highest GDH, and vice-versa. In Portugal, the fresh fruit region with the highest heat forcing values is “Algarve” (∼95 GDH), which mainly produces oranges, whereas the lowest value (∼68 GDH) is found in “Beira Alta,” a region that typically produces apples.

### Future Projections

The climate change projections for SWC reveal a strong decrease (Figure 2A), pointing to generalized warming throughout Portugal. This reduction is noticeably stronger under RCP8.5 and more pronounced for the latter sub-periods (2041–2060 and 2061–2080), which are subjected to stronger anthropogenic forcing. For RCP4.5, the reduction is limited to -12 CP until the last period. Conversely, for the more severe scenario, the reduction in chilling can be larger than -24 CP. In fact, the reduction of chilling is expected to be much stronger in inner Portugal (<-20 CP for RCP8.5) than in southern coastal areas.

Concerning SHF (Figure 2B), this index reveals an increase over most of Portugal, particularly over the northern and western areas (up to +15 GDH under RCP8.5). The increase in GDH does not linearly reflect the upward trend in temperature, due to the critical threshold in heat accumulation from this index definition. Although, it is expected that in some areas the increase in GDH is not very noticeable, the overall warming can be several times higher, due to this upper temperature threshold. Conversely, some areas in the cooler northern regions may benefit from future warming, owing to the more frequent nearly optimum temperatures for fruit growth (25°C), while southern areas, which are already considered very hot, may become excessively warm, with more frequent above-optimum temperatures. Hence, the northeast-south gradient in heat accumulation over Portugal should be reduced under future climates.

**FIGURE 4:**
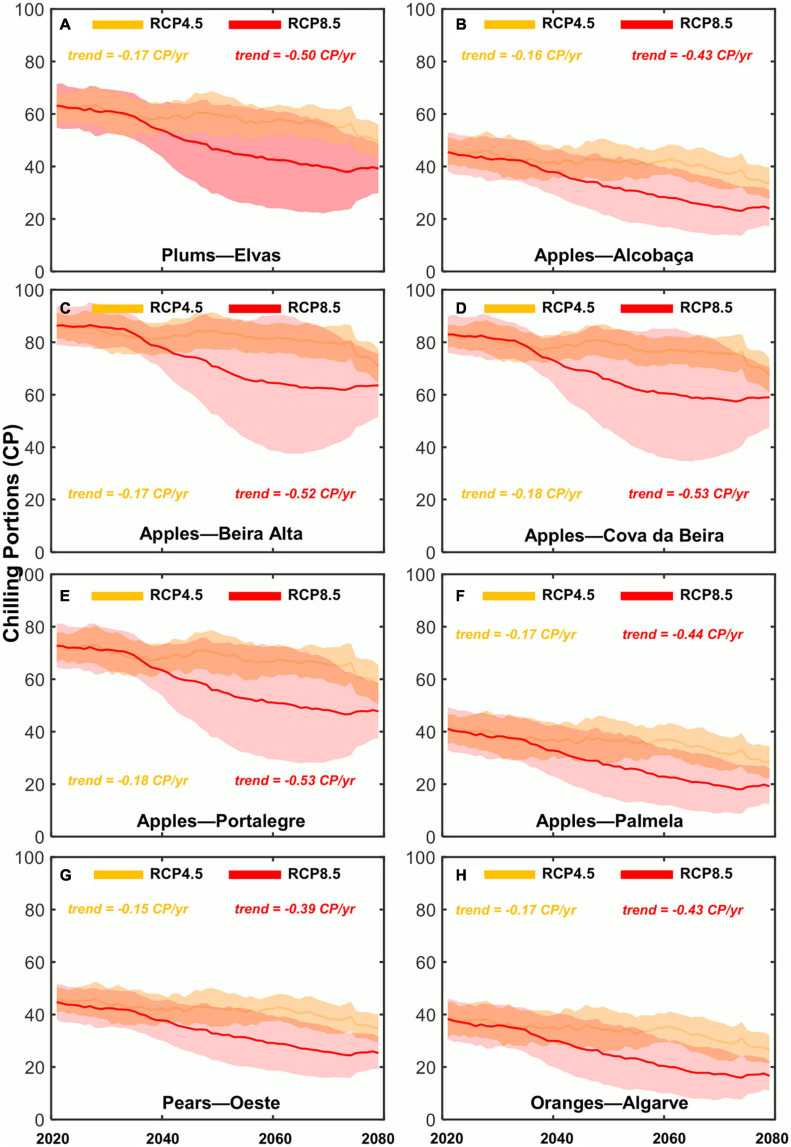
Chronograms (2020–2080) of the Chilling Portions (CP) index for the two future scenarios (RCP4.5 and RCP8.5) over the selected eight denominations of origin regions in mainland Portugal: **(A)** Plums—Elvas; **(B)** apples—Alcobaça; **(C)** apples—Beira Alta; **(D)** apples—Cova da Beira; **(E)** apples—Portalegre; **(F)** apples—Palmela; **(G)** pears—Rocha do Oeste; **(H)** oranges—Algarve. Thick lines represent the climate model ensemble means of each RCP as a function of time, while shaded areas represent the corresponding climate model standard deviations.

**FIGURE 5:**
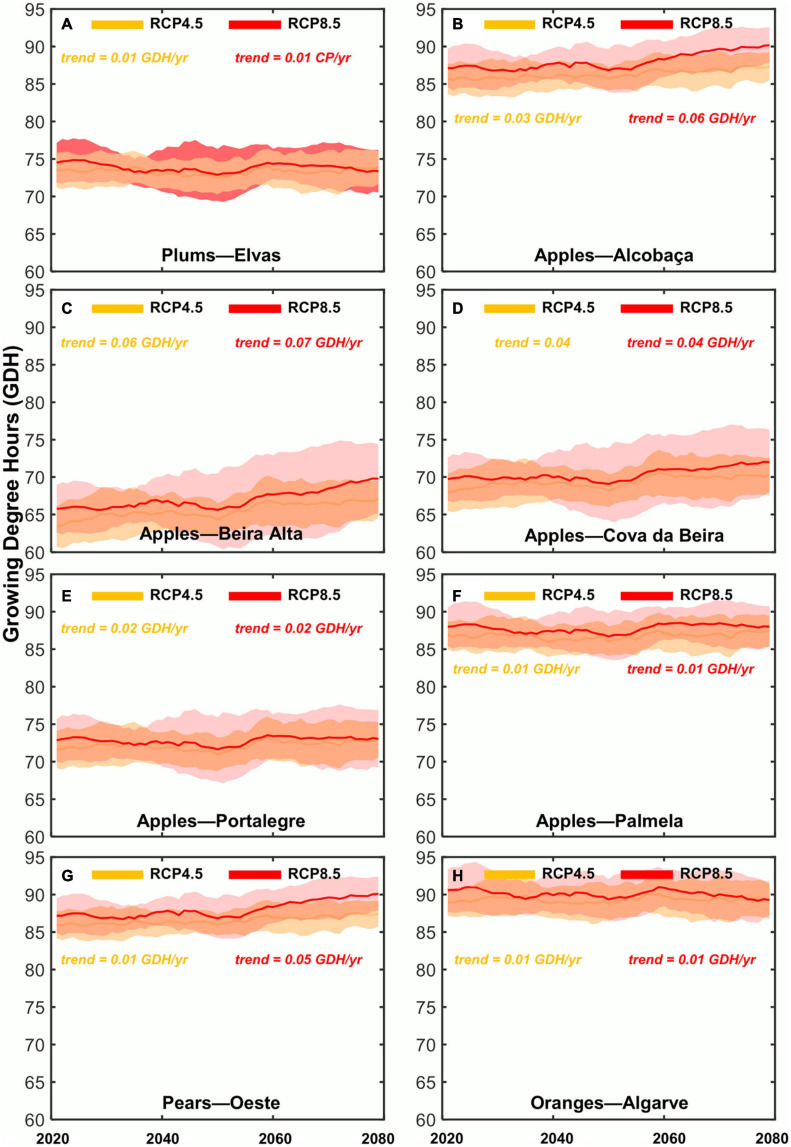
Same as Figure 4 but for the Growing Degree Hour (GDH) index. Values are shown in ×10^3^.

With respect to the interannual variability and CP-GDH evolution under future climates, it is consistent with the subperiod mean (Figures 4, 5). Overall, the CP shows a decreasing trend until 2080, while the GDH shows an increasing trend, although less pronounced. For the CP, under RCP4.5, future liner trends (LT) for the fresh fruit regions range from LT = −0.15 to −0.18 CP/year. For RCP8.5, LT range from −0.39 to −0.53 CP/year. For the GDH increasing future trend are found, which are quite similar for the two future scenarios, range from LT = 0.01 to 0.05 GDH/year. Also in Figures 4, 5, the climate model agreement and uncertainties can be analyzed. It becomes clear that the climate change signal of both chill accumulation and heat forcing is also very robust. The model standard deviation (spread) shows the same trends as the climate model ensemble mean. Generally, RCP4.5 shows a higher agreement amongst models than RCP8.5, particularly after 2050. The climate model standard deviation from 2050 onward is relatively large, which indicates a strong model uncertainty for this index. These uncertainties are also stronger for the regions in the innermost areas of the country, which tend to be apple-producing areas. For the GDH model standard deviation is similar across all regions and future climatic scenarios.

**FIGURE 6:**
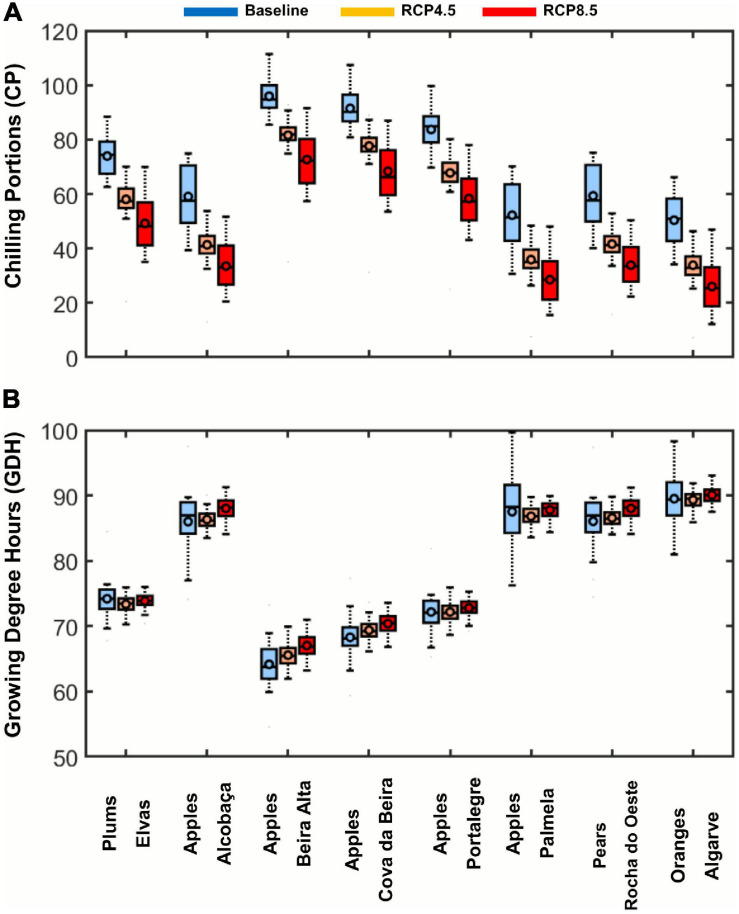
Boxplots of the distribution of **(A)** Chilling Portions (CP) and **(B)** Growing Degree Hours (GDH), for baseline and two future scenarios (RCP4.5 and RCP8.5), and by the denomination of origin regions. Each box represents the 25th and 75th quantiles, the inner circle the mean, and the inner line the median. The outer whiskers represent the maxima and minima. GDH values are shown in ×10^3^.

Figure 6 shows that, regionally, Portugal is expected to undergo important climatic changes for temperate fruit trees. For all regions, the chilling accumulation is generally projected to decrease, whereas the heat accumulation is projected to increase, owing to heat stress conditions under much warmer wintertime and spring-summer periods. From the regional analysis under future climate, it is clear that some apple-, pear-, and plum-producing regions are expected to have chilling conditions similar or even warmer than todays’ orange-producing regions. This is the case of “Elvas” for plums, “Alcobaça,” “Palmela” for apples and “Rocha do Oeste” for pears. Furthermore, under RCP8.5, this enhanced warming during winter may lead to detrimental conditions for several varieties planted in these regions. Regarding forcing, “Beira Alta,” “Cova da Beira,” and “Portalegre,” which are apple-producing regions, will remain as the regions with the lowest GDH values.

## Discussion and Conclusion

The objectives of the present study were to quantify the impacts of climate change on the main fresh fruit regions in Portugal. Chilling and forcing for plants were computed using bias-corrected data from several RCM-GCM model chains. Given the results for the future chilling and forcing in Portuguese fruit orchards, it is evident that climate change may have important implications for these food crops. These results are in agreement with previous studies that suggested that the current fruit production might be particularly vulnerable under future climate (Baldocchi and Wong, 2008; Luedeling et al., 2011).

Portugal will undergo strong declines in winter chilling. The generalized decrease in SWC will more severely affect the inner-most regions of the country, in agreement with several studies (Luedeling and Brown, 2011; Luedeling et al., 2011; Fraga et al., 2019). Important fruit regions are affected by this change, and the more chill demanding trees, such as plum, apple, or pear (Luedeling et al., 2013; Morais and Carbonieri, 2015), may indeed suffer the strongest impacts. The northern apple growing areas are particularly exposed to chilling reduction, evidencing the significance of the current study for supporting effective climate change adaptation measures of fresh fruit crops. Higher winter temperatures may be detrimental, as insufficient chilling may cause delayed budding and foliation, resulting in low fruit-set/yields (Petri and Leite, 2004). Furthermore, a stronger inter-annual chill variability may lead to new problems (Ghrab et al., 2014), while the lack of proper winter chill may increase bud abscission, causing growth anomalies (Petri and Leite, 2004). For other less chill demanding fruit trees *or trees with no chilling requirments*, such as oranges, the projected chilling should not affect a proper flowering and fruit-set, and a more positive response to this warming is expected (Guo et al., 2015a). This fruit/region should benefit from the projected warming, as warmer conditions tend to benefit crop yields (Tubiello et al., 2002). Nonetheless, it is important to note that this study does not consider other climate change-related aspects, such as water availability in the future.

Regarding forcing, the ensemble projections indicate that Portugal will experience enhanced warming during the plant developmental stage, with a very high agreement amongst the 4-GCM-RCM models. The generalized increase in SHF will tend to affect more severely the southern and coastal areas of the country. A higher forcing during the growing season should result in earlier phenological stages with multiple implications, as the temperature plays a key role in determining phenology (El Yaacoubi et al., 2020). As an example, earlier bloom could potentially coincide with spring frost, resulting in damage to flowers. Earlier flowering may also reduce the pollination periods and enhance plant competition for nutrients and water (Lorite et al., 2020). Additionally, higher temperatures during ripening may result in faster and unbalanced fruit maturity, which may lead to implications on fruit quality, fruit-set, and yields (Campoy et al., 2011; Luedeling et al., 2011). It should be noted that the effect of extreme weather events such as heatwaves are not taken into account by these indices, and are expected to become a major thread for the Mediterranean orchards under future cliamtes (Fraga et al., 2020a).

The present study outcomes suggest a loss of suitability of some current fruticulture areas, owing to lower chilling during autumn-winter and warming during spring–summer, particularly in innermost areas of the country. Climate change impacts will be closely tied to the fruit trees growing in a given region (Luedeling et al., 2011; Atkinson et al., 2013). It is important to note that the present findings are also crop- and variety-dependent, and may not be directly compared given the specificities of each crop and variety (Guo et al., 2015a). However, timely planning of suitable adaptation measures may help mitigating future yield/quality losses and warrant the future sustainability of this sector. Crop relocation, though possible, is a long-term measure and is far from being an ideal solution. Hence, to deal with this regional climatic change, it becomes crucial to develop and implement suitable cultural practices designed to deal with specific climate change threats.

Regarding the lower chilling, the focus must be given to the adoption of less chill-demanding varieties or clones. The current study allows to differentiate between fresh fruit regions and the varieties grown therein, i.e., different types of apples are grown at each of the regions, each with different chilling requirements. The availability of varieties with lower chilling requirements may increase the future sustainability of this sector and is certainly a suitable adaptation option for the most affected areas. It is clear that under future climates and lower overall chilling, some of these varieties will tend to migrate to other regions, depending on the regional climate conditions. The importance of variety selection for crop adaptation has been highlighted by several studies (Soloklui et al., 2017; Rodriguez et al., 2019).

Nonetheless, it is projected that under the most severe scenario (RCP8.5), the reduction in SWC should be much more intense. Furthermore, taking into account model uncertainties, some models predict a very strong reduction of CP, with a higher interannual variability, more pronounced after 2050, in agreement with other studies (Rodriguez et al., 2021). It becomes clear that in the Mediterranean areas, which are indeed a climate change hot spot, there can be difficulties fulfilling apple chilling requirements under future climatic conditions (Funes et al., 2016; Fernandez et al., 2020). For these cases, it is expected that changing varieties may not be sufficient, and other adaptation options should be envisioned. The development of practices to artificially break dormancy can also be considered (Luedeling et al., 2011). Other cultural practices, such as the improvement and adoption of appropriate scion-rootstock combinations, or even targueted defoliation may also potentially reduce chilling requirements.

To deal with excessive heat during spring-summer, more focus should be given to plant thermal stress. The implementation of deficit-irrigation strategies might be a suitable adaptation strategy under future warmer climates (Lorite et al., 2020). Additionally, the reduction of excessive heat and radiation fluxes can be achieved through the application of sunscreens, or certain types of shading systems, which may influence orchard microclimates (Glenn et al., 2002; Lopez et al., 2018). Furthermore, more heat-tolerant plant varieties and more adapted varieties should also be selected. Nonetheless, taking into account the degrees of severity of the different climate change scenarios, these adaptation measures may not be sufficient, e.g., under RCP8.5, and crop relocation may also be necessary. Certain subtropical or tropical fruits are naturally adapted to warm climates and may replace some current temperate fruit trees currently grown in Portugal. Nonetheless, growers will play a central role in this decision-making process toward climate change adaptation and risk reduction. Besides, the adoption of these measures requires further research and planning to ensure the future economic sustainability of fruit orchards in Portugal. As a final remark, this study results support that local adaptation will be much more feasible under a moderate warming scenario (RCP4.5), which is yet another reason to limit the warming rate within the 1.5°C, as recommended by the Paris Agreements and the last IPCC special report (IPCC, 2019).

It should be pointed out that, that under future climates the relationship between chilling and forcing may not be linear and these two factors are not independent of each other. A higher forcing under climate change does not necessarily result in earlier phenological stages, since warmer winters may result in delayed chilling fulfillment. Furthermore, chilling and forcing have a compensation effect, by which some chill beyond a minimum requirement can lower the amount of heat required for plants (Pope et al., 2014). Therefore, additional studies are necessary to address the issue of lower chilling and higher forcing for plants under future climate scenarios.

## Data Availability Statement

The original contributions presented in the study are included in the article/Supplementary Material, further inquiries can be directed to the corresponding author.

## Author Contributions

Both authors have contributed to this article and agreed with the contents.

## Conflict of Interest

The authors declare that the research was conducted in the absence of any commercial or financial relationships that could be construed as a potential conflict of interest.
