# Supplementary material for: Assessment of Climate Change Impacts on Chilling and Forcing for the Main Fresh Fruit Regions in Portugal
Source: Front Plant Sci. 2021 Jun 23;12:689121. doi: 10.3389/fpls.2021.689121 (PMC8262527; doi:10.3389/fpls.2021.689121)
Supplement: Supplementary file 1 [file Data_Sheet_1.PDF]

Assessment of climate change impacts on chilling and heat forcing for the main fresh fruit trees in Portugal

Helder Fraga[a,b,1]; João A. Santos[a,b]

[a]*Centre for the Research and Technology of Agro-Environmental and Biological Sciences, CITAB, Universidade de Trás-os-Montes e Alto Douro, UTAD, 5000-801 Vila Real, Portugal*

[b]*Institute for Innovation, Capacity Building and Sustainability of Agri-food Production*

Supplementary Material

[1]*The corresponding author*: Helder Fraga, E-mail: hfraga@utad.pt

Table S1 – Global climate models (GCM) and regional climate models (RCM) used in this study.

GCM	RCM
MPI-M-MPI-ESM-LR	CLMcom-CCLM4-8-17
IPSL-IPSL-CM5A-MR	IPSL-INERIS-WRF331F
ICHEC-EC-EARTH	KNMI-RACMO22E
CNRM-CERFACS-CNRM-CM5	SMHI-RCA4

```
%   Automate the ChillR R script spatially - by Helder Fraga
 <hfraga@utad.pt>

%   This program is free software: you can redistribute it and/or
 modify
%   it under the terms of the GNU General Public License as published
 by
%   the Free Software Foundation, either version 3 of the License, or
%   (at your option) any later version.
%
%   This program is distributed in the hope that it will be useful,
%   but WITHOUT ANY WARRANTY; without even the implied warranty of
%   MERCHANTABILITY or FITNESS FOR A PARTICULAR PURPOSE.  See the
%   GNU General Public License for more details.

%   You should have received a copy of the GNU General Public License
%   along with this program.  If not, see <https://www.gnu.org/
licenses/>
%


clc
clear all, close all

% set start and end dates

startday = '2021/01/01';
endday = '2080/12/31';

rcm = 'MPI-CSC-REMO2009'; % KNMI-RACMO22E DMI-HIRHAM5 SMHI-RCA4
gcm = 'MPI-M-MPI-ESM-LR'; % ICHEC-EC-EARTH MOHC-HadGEM2-ES MPI-M-MPI-
ESM-LR
scn = 'rcp45';

year1 = year(startday);
year2 = year(endday);
nyears = year2 - year1 +1;
skip = 0;
skip2 = 0;

currentdir = strcat (pwd , '\');
datadir = strcat('F:\CORDEX025_correct\',rcm,'\' ,gcm,'\' , scn, '\');
factorsfolder = strcat('F:\CORDEX025_correct\',rcm,'\' ,gcm,'\FACTORS
\');


rdir1 = strcat('F:/CORDEX025_correct/',rcm,'/' ,gcm,'/' , scn, '/
in/');
rdir2 = strcat('F:/CORDEX025_correct/',rcm,'/' ,gcm,'/' , scn, '/
out_chill/');
rdir3 = strcat('F:/CORDEX025_correct/',rcm,'/' ,gcm,'/' , scn, '/
out_gdh/');
```

```
% read climate data netcdfs

disp('read tn');
f= strcat(datadir,'tasmax.nc');
nc=netcdf.open(f,'NC_NOWRITE');
vartlat = netcdf.inqVarID(nc,'lat');
vartlon = netcdf.inqVarID(nc,'lon');
varttime = netcdf.inqVarID(nc,'time');
varttx = netcdf.inqVarID(nc,'tasmax');
lon = netcdf.getVar(nc,vartlon);
lat = netcdf.getVar(nc,vartlat);
time = netcdf.getVar(nc,varttime);
nlat=length(lat);nlon=length(lon);nt = length(time);
tx = double(netcdf.getVar(nc,varttx));
netcdf.close(nc);

disp('read tx');
f= strcat(datadir,'tasmin.nc');
nc=netcdf.open(f,'NC_NOWRITE');
vartlat = netcdf.inqVarID(nc,'lat');
vartlon = netcdf.inqVarID(nc,'lon');
varttime = netcdf.inqVarID(nc,'time');
varttn = netcdf.inqVarID(nc,'tasmin');
lon = netcdf.getVar(nc,vartlon);
lat = netcdf.getVar(nc,vartlat);
time = netcdf.getVar(nc,varttime);
nlat=length(lat);nlon=length(lon);nt = length(time);
tn = double(netcdf.getVar(nc,varttn));
netcdf.close(nc);



tx(tx == -9999)= nan;
tn(tn == -9999)= nan;
tx = tx -273.15;
tn = tn -273.15;
if skip2 ~= 1

if skip ~= 1

    status = dos(strcat('md',32,datadir,'in'));
    status = dos(strcat('md',32,datadir,'out_chill'));
    status = dos(strcat('md',32,datadir,'out_gdh'));

    status = dos(strcat('del',32,datadir,'in\*.*'));
    status = dos(strcat('del',32,datadir,'out_chill\*.*'));
    status = dos(strcat('del',32,datadir,'out_gdh\*.*'));

basis =0;
if nt == daysact(startday, endday) +1
    disp('366 day calendar');
    basis = 0;
end
```

```
if nt == days365(startday, endday) +1
    disp('365 day calendar');
    basis = 3;
end
if nt == days360(startday, endday) +1
    basis = 2;
end


for t = 1:nt
    M(t,:) = datevec(daysadd(startday, t-1, basis));
end

D=M(:,1:3);


disp('Write input files');

for lt = 1:nlat
    disp (lt);
    for ln = 1:nlon
        D(:,4)=squeeze(tx (ln,lt,:));
        D(:,5)=squeeze(tn (ln,lt,:));
        filename = strcat(num2str(lt),'_',num2str(ln),'.csv');
        if (isnan(tn(ln,lt,1)) || isnan(tx(ln,lt,1)))
            %a
        else
% %             tic;
            fid = fopen(strcat(datadir,'in\',filename), 'w'); ...
            fprintf(fid,'%d,%d,%d,%f,%f\n',D'); ...
            fclose(fid);
%
%              csvwrite (strcat(datadir,'in\',filename),D);
% % toc;
        end
    end
end

end


% run ChillR scripts outside matlab and read outputs from files
disp('Run ChillR');
status = dos(strcat('Rscript.exe',32, currentdir,'chill_calculator.R
 ',32, rdir1,' ',32, rdir2));
pause(3);

status = dos(strcat('Rscript.exe',32, currentdir,'gdh_calculator.R
 ',32, rdir1,' ',32, rdir3));
pause(3);
end
disp('Read output files');

OUT_chill = nan(nlon,nlat,nyears);
```

```
for lt = 1:nlat
    disp (lt);
    for ln = 1:nlon
        if (isnan(tn(ln,lt,1)) || isnan(tx(ln,lt,1)))
            %a
        else
            filename = strcat(num2str(lt),'_',num2str(ln),'.csv');
            out = csv2cell(strcat(datadir,'out_chill
\',filename), 'fromfile');
            out1 = out(2:length(out),10);
            out2 = str2double(out1);
            OUT_chill (ln,lt,1:length(out2)) = out2;
        end
    end
end



 % write outputs of rscript to a netcdf
OUT_chill_mean = nanmean(OUT_chill,3);

outFile= strcat(datadir,rcm,'_',scn,'_chill_mean.nc');
ncid = netcdf.create(outFile,'NC_SHARE');
dimid0 = netcdf.defDim(ncid,'lon',nlon);
dimid1 = netcdf.defDim(ncid,'lat',nlat);
varid0 = netcdf.defVar(ncid,'lon','double',dimid0);
varid1 = netcdf.defVar(ncid,'lat','double',dimid1);
varid2 = netcdf.defVar(ncid,'chill','double',[dimid0 dimid1]);
netcdf.putAtt(ncid,varid0,'long_name','longitude')
netcdf.putAtt(ncid,varid0,'units','degrees_east')
netcdf.putAtt(ncid,varid0,'standard_name','longitude')
netcdf.putAtt(ncid,varid0,'_CoordinateAxisType','Lon')
netcdf.putAtt(ncid,varid1,'long_name','latitude')
netcdf.putAtt(ncid,varid1,'units','degrees_north')
netcdf.putAtt(ncid,varid1,'standard_name','latitude')
netcdf.putAtt(ncid,varid1,'_CoordinateAxisType','Lat')
netcdf.endDef(ncid);
netcdf.putVar(ncid,varid0,lon);
netcdf.putVar(ncid,varid1,lat);
netcdf.putVar(ncid,varid2,OUT_chill_mean);
netcdf.close(ncid);


outFile= strcat(datadir,rcm,'_',scn,'_chill_yearly.nc');
ncid = netcdf.create(outFile,'NC_SHARE');
dimid0 = netcdf.defDim(ncid,'lon',nlon);
dimid1 = netcdf.defDim(ncid,'lat',nlat);
dimid2 = netcdf.defDim(ncid,'time',nyears);
varid0 = netcdf.defVar(ncid,'lon','double',dimid0);
varid1 = netcdf.defVar(ncid,'lat','double',dimid1);
varid2 = netcdf.defVar(ncid,'time','double',dimid2);
varid3 = netcdf.defVar(ncid,'chill','double',[dimid0 dimid1 dimid2]);
netcdf.putAtt(ncid,varid0,'long_name','longitude')
```

```
netcdf.putAtt(ncid,varid0,'units','degrees_east')
netcdf.putAtt(ncid,varid0,'standard_name','longitude')
netcdf.putAtt(ncid,varid0,'_CoordinateAxisType','Lon')
netcdf.putAtt(ncid,varid1,'long_name','latitude')
netcdf.putAtt(ncid,varid1,'units','degrees_north')
netcdf.putAtt(ncid,varid1,'standard_name','latitude')
netcdf.putAtt(ncid,varid1,'_CoordinateAxisType','Lat')
netcdf.endDef(ncid);
netcdf.putVar(ncid,varid0,lon);
netcdf.putVar(ncid,varid1,lat);
netcdf.putVar(ncid,varid2,1:nyears);
netcdf.putVar(ncid,varid3,OUT_chill);
netcdf.close(ncid);


OUT_gdh = nan(nlon,nlat,nyears);

for lt = 1:nlat
    disp (lt);
    for ln = 1:nlon
        if (isnan(tn(ln,lt,1)) || isnan(tx(ln,lt,1)))
            %a
        else
            filename = strcat(num2str(lt),'_',num2str(ln),'.csv');
            out = csv2cell(strcat(datadir,'out_gdh
\',filename), 'fromfile');
            out1 = out(2:length(out),11);
            out2 = str2double(out1);
            OUT_gdh (ln,lt,1:length(out2)) = out2;
        end
    end
end

OUT_gdh_mean = nanmean(OUT_gdh,3);

outFile= strcat(datadir,rcm,'_',scn,'_gdh_mean.nc');
ncid = netcdf.create(outFile,'NC_SHARE');
dimid0 = netcdf.defDim(ncid,'lon',nlon);
dimid1 = netcdf.defDim(ncid,'lat',nlat);
varid0 = netcdf.defVar(ncid,'lon','double',dimid0);
varid1 = netcdf.defVar(ncid,'lat','double',dimid1);
varid2 = netcdf.defVar(ncid,'gdh','double',[dimid0 dimid1]);
netcdf.putAtt(ncid,varid0,'long_name','longitude')
netcdf.putAtt(ncid,varid0,'units','degrees_east')
netcdf.putAtt(ncid,varid0,'standard_name','longitude')
netcdf.putAtt(ncid,varid0,'_CoordinateAxisType','Lon')
netcdf.putAtt(ncid,varid1,'long_name','latitude')
netcdf.putAtt(ncid,varid1,'units','degrees_north')
netcdf.putAtt(ncid,varid1,'standard_name','latitude')
netcdf.putAtt(ncid,varid1,'_CoordinateAxisType','Lat')
netcdf.endDef(ncid);
netcdf.putVar(ncid,varid0,lon);
netcdf.putVar(ncid,varid1,lat);
netcdf.putVar(ncid,varid2,OUT_gdh_mean);
```

```
netcdf.close(ncid);


outFile= strcat(datadir,rcm,'_',scn,'_gdh_yearly.nc');
ncid = netcdf.create(outFile,'NC_SHARE');
dimid0 = netcdf.defDim(ncid,'lon',nlon);
dimid1 = netcdf.defDim(ncid,'lat',nlat);
dimid2 = netcdf.defDim(ncid,'time',nyears);
varid0 = netcdf.defVar(ncid,'lon','double',dimid0);
varid1 = netcdf.defVar(ncid,'lat','double',dimid1);
varid2 = netcdf.defVar(ncid,'time','double',dimid2);
varid3 = netcdf.defVar(ncid,'gdh','double',[dimid0 dimid1 dimid2]);
netcdf.putAtt(ncid,varid0,'long_name','longitude')
netcdf.putAtt(ncid,varid0,'units','degrees_east')
netcdf.putAtt(ncid,varid0,'standard_name','longitude')
netcdf.putAtt(ncid,varid0,'_CoordinateAxisType','Lon')
netcdf.putAtt(ncid,varid1,'long_name','latitude')
netcdf.putAtt(ncid,varid1,'units','degrees_north')
netcdf.putAtt(ncid,varid1,'standard_name','latitude')
netcdf.putAtt(ncid,varid1,'_CoordinateAxisType','Lat')
netcdf.endDef(ncid);
netcdf.putVar(ncid,varid0,lon);
netcdf.putVar(ncid,varid1,lat);
netcdf.putVar(ncid,varid2,1:nyears);
netcdf.putVar(ncid,varid3,OUT_gdh);
netcdf.close(ncid);
```

```
Undefined function 'year' for input arguments of type 'char'.

Error in source_code_chillr_cp_gdh (line 30)
year1 = year(startday);
```

Published with MATLAB® R2020b
